# An evaluation of Croí MyAction community lifestyle modification programme compared to standard care to reduce progression to diabetes/pre-diabetes in women with prior gestational diabetes mellitus (GDM): study protocol for a randomised controlled trial

**DOI:** 10.1186/1745-6215-14-121

**Published:** 2013-05-02

**Authors:** Jennifer J Infanti, Fidelma P Dunne, Angela O’Dea, Paddy Gillespie, Irene Gibson, Liam G Glynn, Eoin Noctor, John Newell, Brian E McGuire

**Affiliations:** 1 School of Medicine, Clinical Sciences Institute, National University of Ireland Galway, Galway, Ireland; 2 J.E. Cairnes School of Business & Economics, Cairnes Building, National University of Ireland Galway, Galway, Ireland; 3 Croí–The West of Ireland Cardiac Foundation, Croí House, Moyola Lane, Newcastle, Galway, Ireland; 4 Discipline of General Practice, School of Medicine, National University of Ireland Galway, 1 Distillery Road, Galway, Ireland; 5 HRB Clinical Research Facility Galway, National University of Ireland Galway, University Road, Galway, Ireland; 6 School of Psychology, National University of Ireland Galway, University Road, Galway, Ireland

**Keywords:** Gestational diabetes mellitus, Pre-diabetes, Risk factor modification, Lifestyle intervention, Interdisciplinary approach, Randomised controlled trial

## Abstract

**Background:**

Universal screening using the International Association of Diabetes and Pregnancy Study Groups (IADPSG) criteria has identified a prevalence of gestational diabetes mellitus (GDM) of 12.4% in women living in Ireland. Women with prior GDM are at increased risk of developing type 2 diabetes later in life. A number of risk factors linked to the development of type 2 diabetes are potentially modifiable through lifestyle and behaviour changes, and medical management. No previous Irish studies have adequately investigated the efficacy of lifestyle intervention programmes in reducing these risk factors in women with prior GDM. Through a two-group, parallel randomised controlled trial (RCT), this study aims to assess the clinical impact, cost-effectiveness and psychological experience of the Croí MyAction intensive lifestyle modification programme for women with prior GDM.

**Methods/Design:**

A total of 54 women with a history of GDM and persistent post-partum glucose dysfunction (impaired glucose tolerance (IGT) or impaired fasting glucose (IFG)), are randomly assigned to a control arm (n = 27) or to the Croí MyAction intervention group (n = 27). The control arm receives usual health care advice - written information on diet and lifestyle changes for reducing diabetes risks and visits with general practitioners as required. The intervention group receives usual health care as per the control group in addition to attending a 12-week intensive lifestyle modification programme known as Croí MyAction. Croí MyAction involves 2.5 hour sessions once per week (for 12 weeks) comprising a group exercise programme, group health promotion or education seminars, and one-to-one meetings with a multidisciplinary health care team to personalise risk factor reductions. Randomisation and allocation to the intervention arms is carried out by an independent researcher, ensuring that the allocation sequence is concealed from study researchers until the interventions are assigned. The primary analysis is based on glucose dysfunction, comparing a mean reduction in fasting plasma glucose (FPG) levels on a 75 gram oral glucose tolerance test (OGTT) in the two groups at a one-year, post-intervention follow-up. The trial is funded by the Irish Health Research Board (HRB). Ethics approval was obtained on 27 March 2012 from the Clinical Research Ethics Committee, Galway University Hospitals, Health Service Executive of Ireland (Ref: C.A.691).

**Trial registration:**

Current Controlled Trials ISRCTN41202110.

## Background

Gestational diabetes mellitus (GDM) is defined as any degree of glucose intolerance resulting in hyperglycaemia, or excess sugar, in a mother’s blood during pregnancy [1]. When present, the pregnancy may be associated with a number of adverse maternal and neonatal outcomes. Neonatal complications include hypoglycaemia respiratory distress syndrome and jaundice, and macrosomia may result in shoulder dystocia [2-5]. Adverse maternal outcomes include pregnancy induced hypertension (PIH), pre-eclampsia toxaemia (PET), polyhydramnios and an increased risk of delivery by caesarian section [2,4-6]. GDM is also associated with increased psychological stress during pregnancy [7]. Later in life, both the mother and child are at increased risk of hospital admission, obesity, type 2 diabetes and heart disease [8-14].

There is a paucity of robust evidence relating to the prevalence of GDM in the international literature [15]. Prevalence estimates have been complicated by the wide range of definitions and test criteria used for diagnosing GDM and vary according to region and ethnic group [16]. There is a general consensus, however, that the prevalence of GDM is increasing globally and occurs in approximately 2 to 9% of all pregnancies [6,17]. Several factors have been shown to be associated with an increased risk of GDM. Increasing age, previous GDM, pre-pregnancy obesity, a previous macrosomic baby (larger than 4.5 kg), a family history of diabetes (amongst first-degree relatives), and particular ethnic origins are generally accepted as risk factors [16]. In Ireland, accurate and up-to-date data on the incidence of hyperglycaemia in pregnancy were lacking prior to 2007, when the first results of the Atlantic Diabetes in Pregnancy (DIP) research programme were published. The Atlantic DIP programme, funded between 2005 and 2010 by Ireland’s Health Research Board, found that at least 12% of pregnancies are complicated by GDM in Ireland [3]. In addition, approximately 18% of these women suffered from persistent post-partum glucose abnormalities in the first year following the birth, compared to 2% in women with proven normal glucose tolerance (NGT) in the index pregnancy, during the same time and accessing the same health care services in the same geographical location [13]. These glucose abnormalities included type 2 diabetes, but also IFG, IGT, metabolic syndrome (MetS) and insulin resistance (IR), conditions recognised as intermediary stages in the development of type 2 diabetes.

Diabetes is associated with significant morbidity and mortality and is one of the fastest growing chronic health conditions in Ireland and worldwide. Some of the biological risk factors associated with the development of type 2 diabetes are non-modifiable, such as age, ethnicity or family history of the disease. However, there are a range of risk factors that are potentially modifiable through lifestyle changes, for example, central adiposity, elevated fasting blood sugars, triglycerides or blood pressure; behavioural factors, such as poor diet or sedentary lifestyle; and a host of cognitive and mood factors which delimit or facilitate healthy lifestyle changes (self-efficacy, motivation to change, depression or anxiety) [18]. A growing body of research suggests that “for people with pre-diabetes, weight loss and therefore reduction in body mass index (BMI) and waist circumference [alone] is successful in reducing the risk of developing diabetes” [18]. Saaristo *et al.* (2010) found that modest weight loss (circa 5%) in a community care setting is especially effective in reducing risk of diabetes [19].

With this evidence, randomised clinical trials in a number of countries have investigated the effects of lifestyle interventions in high risk groups, including women with prior GDM, on the reduction of progression to type 2 diabetes [18,20-23]. These studies have shown that type 2 diabetes can be prevented, or at least postponed, by even modest lifestyle changes. In Ireland, no previous research has adequately evaluated the outcomes of lifestyle intervention for this cohort of women. Without such research, we are left with an inadequate analysis of the efficacy of lifestyle intervention programmes for delaying diabetes onset in women with prior GDM in Ireland. It is also unclear whether participation in such interventions can help women sustain improvements in lifestyle practices, such as diet and physical activity, over time (that is, when the intervention is over). This study seeks to remedy this gap through the development of a randomised controlled trial (RCT) which will evaluate the effectiveness of Croí MyAction, an intensive 12-week lifestyle intervention programme, as compared with standard health care provided to women with a history of GDM. The trial will compare the two study arms in terms of a mean reduction in fasting blood glucose dysfunction from the time of a baseline assessment to a one-year follow-up.

Croí is the West of Ireland Cardiac Foundation. Croí MyAction is a lifestyle and risk factor modification intervention programme offered to people at risk of cardiovascular disease. It is the only initiative of its kind in the country that integrates the care of patients at high risk of cardiovascular diseases and type 2 diabetes in a community-based setting. Croí MyAction was developed in collaboration with the Irish Health Service Executive (HSE) and Imperial College London. The 12-week intervention programme is modeled on the European Society of Cardiology demonstration project, a large clinical trial called EuroAction. EuroAction was a cluster RCT of a preventive cardiology intervention programme in Europe. It demonstrated that nurse-managed, multi-disciplinary, family-based programmes could achieve healthier lifestyle changes and better risk factor control than usual care at the time of a one-year follow-up [24]. The 12-week Croí MyAction programme in Ireland incorporates the principles and protocols of EuroAction. It is delivered by a multidisciplinary team of nurses, dieticians, physiotherapists and physical activity specialists, supported by a physician. It is family-centered, encouraging patients to participate in the programme with a partner. The emphasis of Croí MyAction is on healthy lifestyle change aimed at improving quality and enjoyment of life; for example, empowering individuals and families to make sustainable changes through behavioural approaches, such as motivational interviewing and goal-setting. The programme includes management of blood pressure, lipids, glucose and adherence with cardio-protective medications; finally, it is community-based, thus increasing its accessibility.

While this model of prevention has been evaluated through the EuroAction study, it has not been assessed in women with prior GDM. In this study, therefore, the effectiveness of the Croí MyAction programme will be measured in terms of a reduction in the modifiable biomedical, anthropometric and psychosocial risk factors associated with the development of type 2 diabetes in women with prior GDM. In addition, following the RCT, qualitative methods will be used to examine participants’ experiences of the Croí MyAction intervention programme in terms of its feasibility and perceived strengths and weaknesses. Finally, an economic evaluation will be undertaken to explore the cost-effectiveness of the programme. This integrative, multidisciplinary approach - incorporating clinical, psychological and economic evaluations - is expected to make novel contributions to knowledge in the field of diabetes research.

## Methods/Design

### Setting

The study takes place along the Irish Atlantic seaboard, which extends from the north-western corner of Ireland through the west coast and islands, to the city of Galway. The coordinating centre is the School of Medicine at the National University of Ireland, Galway (NUI Galway), involving the academic departments of medicine, obstetrics, general practice, economics and psychology. The intervention programme is delivered by Croí–West of Ireland Cardiac Foundation, a community-based organisation also located in Galway. All data collection, including the baseline tests, the Croí MyAction intervention programme, and the one-year follow-up tests, occur in a community setting. Ethics approval for the study was obtained in March 2012 from the Clinical Research Ethics Committee of Galway University Hospital, part of Ireland’s Health Service Executive (HSE).

### Hypothesis

The formal null hypothesis to be tested in this study is that there is no difference in mean reduction in glucose dysfunction amongst the population of women with prior GDM following participation in Croí MyAction when compared with women receiving usual health care. The alternative hypothesis is that the Croí MyAction group will show significantly better outcomes than women receiving usual care.

### Sample size and power calculation

The sample size required for the RCT is 54 participants. This number was calculated based on estimates provided from pilot data from a group of women with a history of GDM (n = 74) receiving conventional health care. The pilot group had a standard deviation of 0.64 mmol/l for the difference in fasting plasma glucose (FPG) on an oral glucose tolerance test between two time points: approximately three months post-pregnancy and then one to three years later. Given the standard deviation, it is estimated that a sample size of 27 in each study arm is necessary to have 80% power to detect a mean difference of 0.5 mmol/l in FPG between baseline and one-year follow-up in the two study arms, using a two-sample t-test at the 0.05 significance level.

### Participants: recruitment and eligibility

**Figure 1:**
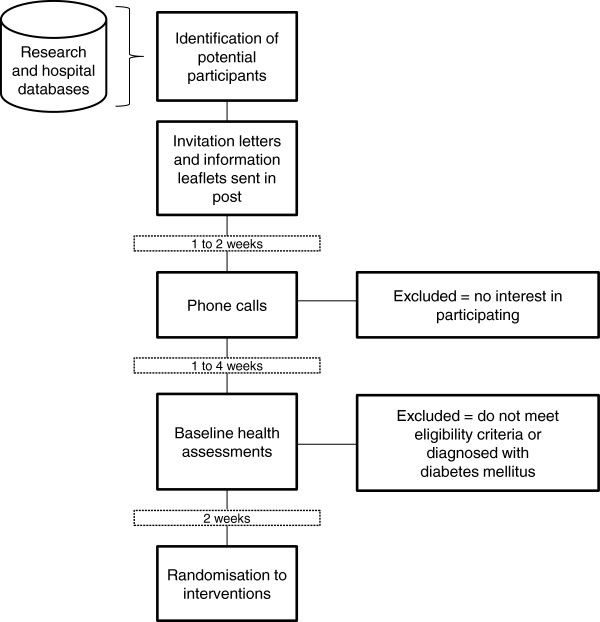
Participant flow through recruitment process.

Potential participants are identified from the Atlantic Diabetes in Pregnancy (DIP) research database and the pregnancy service of the Diabetes Day Centre at the University Hospital Galway. These women are sent invitation letters and research study information leaflets in the post. The mail-outs are then followed by phone calls from the study researchers to determine interest in the study (that is, obtain verbal consent), answer any questions about the study, and schedule appointments for baseline health assessments over the following month. On the baseline assessment days, the researchers obtain written, informed consent from each participant and administer a series of clinical, anthropometric, behavioural and psychological tests, measures and surveys, as described in Table 1 on the secondary outcome measures of the trial. The recruitment process is anticipated to span 10 months, from June 2012 to March 2013. The flow chart in Figure 1 represents the movement of a participant through the stages of recruitment to the study.

**Table 1:** Secondary outcomes: definitions and measurement techniques

	**Outcome**	**Definition and measurement**	**Timepoint measured**		
			**Baseline**	**Post-intervention**	**1-year follow-up**
1	Postprandial glucose dysfunction	Mean reduction in two-hour postprandial (PP) blood glucose on a 75 gram oral glucose tolerance test (OGTT)	✓		✓
2	Insulin resistance	Decrease in insulin resistance as measured by Homeostasis Model Assessment (HOMA) on insulin and glucose values on a 75 gram OGTT	✓		✓
3	Diet adherence	Two-point improvement in overall MedDietScore - a composite diet index based on the traditional Mediterranean dietary pattern	✓	✓	✓
4	Weight and shape	a. Mean weight reduction (kilograms) in individuals with a Body Mass Index (BMI) of >30 kg/m2 at baseline; and	✓	✓	✓
		b. Mean reduction in waist circumference in individuals with a measurement of >88 cm at baseline			
5	Physical activity and fitness	a. Proportions meeting physical activity target of at least 30 minutes of moderate intensity aerobic activity on at least five days of the week; and	✓	✓	✓
		b. Change in functional capacity on an objective physical fitness test (Chester Step Test or six-minute walk test), estimated in metabolic equivalents (METs) or time on test			
6	Lipid profile	a. Proportions achieving a lipid target profile of the following values: total cholesterol <4.5 mmol/l, LDL cholesterol <2.5 mmol/l, triglycerides <1.69 mmol/l, HDL cholesterol >1.29 mmol/l; and	✓	✓	✓
		b. Mean reduction in total and LDL cholesterol			
7	Psychological evaluation (including: positive mental health; general health; quality of life; perceived social support; motivation to change; diabetes-related self-efficacy; depression, anxiety and stress)	a. Positive mental health: four-items from RAND SF-36 questionnaire addressing affective aspects of well-being and five-items from the Mental Health Index-5 (MHI-5) in the RAND SF-36 questionnaire on the occurrence and extent of non-specific psychological distress	✓		✓
		b. General health: Stand-alone measure of self-rated health included in the Irish Survey of Lifestyle, Attitudes and Nutrition (SLÁN), 2007 (originally from The Healthy Days Measures (HRQOL-4))			
		c. Quality of life: Single-item assessment included in SLÁN 2007 (originally from the World Health Organization’s Quality of Life Survey (WHO-QOL Group, 1998))			
		d. Perceived social support: Multidimensional Scale of Perceived Social Support from family, friends, and a significant other (Zimet, Dahlem, Zimet and Farley, 1988)			
		e. Motivation to change: Moore *et al.*’s (2011) scale developed for Australian randomised controlled trial on a lifestyle intervention programme to prevent type 2 diabetes (based on Prochaska *et al.*’s (1992) identification of various stages in relation to maintaining a healthier lifestyle)			
		f. Diabetes-related self-efficacy: 18-item assessment of confidence to engage in exercise and healthy eating under different ‘barrier’ conditions (Moore *et al.*, 2011)			
		g. Depression, anxiety and stress: 21-item Depression, Anxiety and Stress Scale (DASS-21)			

Following the baseline health assessments, eligibility for the study is determined on the following criteria:

#### Inclusion criteria

Participants are women of child-bearing age, 18 years and older, with a history of GDM. At the time of recruitment, they also show at least one of the following diabetes risk factors:

1. Impaired fasting glucose (fasting plasma glucose levels of >5.6 <7 mmol/l^a^); or

2. Impaired glucose tolerance (two-hour plasma glucose levels of >7.8 <11.1 mmol/l); or

3. Insulin resistance based on homeostasis model assessment (HOMA) plus two of the following risk factors: (a) blood pressure > 130/80 mmHG, (b) total cholesterol >4.5 mmol/l, (c) LDL cholesterol >2.5 mmol/l, (d) triglycerides >1.69 mmol/l, (e) HDL cholesterol >1.29 mmol/l, (f) obesity (defined as BMI >30), (g) waist circumference >88 cm.

#### Exclusion criteria

At the time of recruitment, individuals who have any of the following are not eligible to participate in the study: (i) developed type 2 diabetes mellitus; (ii) are currently pregnant; (iii) have inadequate English language ability to understand the content of the intervention programme; or (iv) have significant cognitive impairment or mental illness.

### Randomisation process

The randomisation process in this RCT will use random permuted blocks to ensure similar numbers of participants in each intervention arm throughout the trial and equal numbers in each arm by the end of the study. In advance of participant recruitment, an independent researcher is responsible for generating the allocation sequence using the free computer software program, Random Allocation Software (version 1.0.0), M. Saghaei, Isfahan University of Medical Sciences, Isfahan, Iran. The same independent researcher is responsible for assigning participants to the intervention groups. Thus, the allocation sequence is concealed from all study researchers until the interventions are assigned.

### Description of interventions/comparison groups

The study is a two-group, parallel randomised controlled trial. As such, following the baseline assessments, eligible participants are randomly assigned to the intervention and control groups in an equal ratio of 1:1. Those assigned to the intervention group receive the 12-week intensive lifestyle programme, Croí MyAction. The programme includes an initial individualised assessment by the multidisciplinary health care team, followed by 12 sessions of 2.5 hours each per week (delivered in a maximum of 16 weeks^b^). Each of the weekly sessions comprises a one-hour group exercise programme, a group health promotion and education seminar, and one-to-one meetings to review individual health goals with a specialist nurse, physiotherapist and dietician. As previously mentioned, each Croí MyAction participant is invited to enroll a family member, spouse, friend or other partner in the programme with them for support and motivation outside the programme hours. The intervention is conducted in groups of similar sizes on the same site with the same facilitators, thus ensuring standardised delivery.

The control group receives the standard health care advice provided to women with prior GDM. In this study, standard care is defined as: (i) the provision of educational pamphlets for reducing diabetes risks - particularly, healthy eating and regular physical activity; and (ii) care prescribed by a woman’s general practitioner (GP) as required. It is recognised that GP care will vary from individual to individual.

### Outcomes

The primary outcome measure in the study is glucose dysfunction, which will be assessed in terms of a mean reduction in fasting plasma glucose levels from the time of the baseline assessment to the one-year follow-up assessment, on a 75 gram oral glucose tolerance test. Seven secondary outcomes will also be measured at the baseline and one-year follow-up assessments; namely: two-hour postprandial (PP) blood glucose levels on a 75 gram OGTT, insulin resistance, diet adherence, weight and shape, physical activity and fitness, lipid profile, and psychological evaluation. For participants in the Croí MyAction arm of the study, a number of these outcomes will also be measured immediately following the intervention. Table 1 outlines the definitions, measurement techniques and time points for each of the secondary outcomes.

### Analysis plan

#### Quantitative analysis

All participants will be invited back for one-year follow-up tests to evaluate the effects of the Croí MyAction programme on the same biomedical, anthropometric and psychological measures administered at the baseline assessment. These quantitative data will be analysed in a variety of ways, focusing on comparisons within individual participants (that is, before and after the intervention) and between the study arms (that is, comparing the cohort of women who received the Croí MyAction intervention to the control group participants). In the first instance, a number of numerical and graphical summaries will be used to summarise the groups at baseline and the within-participant correlation structure, pre- and post-intervention. A linear mixed model will be used to compare the change in mean FPG values across time, adjusting for variables, such as age and parity, as relevant. Basic statistical analyses will also be performed to compare data collected from participants in the intervention arm at the end of the Croí MyAction programme and at the one-year follow-up appointment to assess the sustainability of any health improvements made through participation in the intervention.

In the case of missing data, an intention-to-treat analysis will be performed using multiple imputations (based on 10 imputations) and a predictive model-based method. In this method, each missing value is replaced by 10 imputed values, which are generated via random draws from the Bayesian posterior distributions arising from regression models based on chained equations. The selection of explanatory variables for inclusion in the final model will be based on a combination of clustering, tree-based methods and variable selection techniques applied to the complete cases and to the imputed data.

The difference in treatment effect when comparing those who received the intervention to the control group participants will be estimated by pooling the results of each ‘complete model’ (that is, the mean sum of pre- and post-intervention values, with imputed values added where necessary) using the Barnard-Rubin adjustment method. The estimated coefficients, standard errors and *P*-values for the intervention-to-control comparison from both complete and imputed analyses will be reported. Model checking will be performed using suitable model diagnostics and residual plots. All statistical analyses will be performed using the software packages R (version 2.11), R Development Core Team, R Foundation for Statistical Computing, Vienna, Austria and Minitab 16 Statistical Software, Minitab Inc., Taipei, China.

#### Qualitative analysis

Following the RCT, qualitative research will be undertaken to explore the perceptions, views and experiences of women who participated in the randomised controlled trial, comparing Croí MyAction participants with those who followed usual care. This work includes an exploration of the barriers and facilitators to maintaining lifestyle changes. Toward this end, semi-structured, one-to-one interviews will be conducted with 20 participants, 10 from the intervention arm and 10 from the control arm, within six months of completion of the Croí MyAction programme. Participants will be purposively selected to ensure that a representative range of characteristics, such as age and diabetes risk factors at the time of recruitment, are taken into account. All interviews will be recorded and transcribed; data analysis will be based on themes derived from the transcripts. NVivo, a qualitative software package developed by QSR International, Melbourne, Austrlia, will be used to facilitate data management and analysis.

#### Economic evaluation

Finally, an economic evaluation, incorporating both cost-effectiveness analysis and cost-utility analysis, will be performed to explore the cost-effectiveness of the Croí MyAction lifestyle modification programme for women with prior GDM. The economic evaluation will be undertaken in a manner consistent with the guidelines issued by the Health and Information Quality Authority in Ireland [25]. Data will be collected throughout the trial on resource use and outcome measures; these will provide the basis for the analysis. Data will be collected via patient questionnaires on: (a) the costs falling on health and social care systems, and (b) the costs falling on individuals and families. Factors that contribute towards health and social care costs include hospital visits, inpatient stays, GP visits, medicines and other treatments. Factors that contribute towards private costs include travel expenses, time input, and child-minding expenses. Unit costs will be used to convert data on resource use to costs of care. Incremental analysis will be used to provide information on the marginal costs and effects of the programme relative to standard care through the calculation of incremental cost-effectiveness ratios. In terms of effectiveness, the cost-effectiveness analysis will incorporate the primary and secondary outcomes considered in the trial. In addition, the cost-utility analysis will consider effectiveness in terms of Quality Adjusted Life Years (QALYs), as estimated from patient responses to the EuroQol EQ-5D measurement tool [26]. Uncertainty in the analysis will be explored using a combination of univariate and multivariate sensitivity analyses and decision uncertainty will be presented using cost-effectiveness acceptability curves.

## Trial status

Enrolment into the study started on 14 June 2012. Recruitment is expected to be complete by March 2013.

## Endnotes

^a^ The glucose levels used to diagnose impaired fasting glucose and impaired glucose tolerance are based on the 2010 recommendations of the American Diabetes Association and the International Association of Diabetes and Pregnancy Study Groups (IADPSG) [27].

^b^ The Croí MyAction programme allows for participants to complete the 12-week curriculum within 16 weeks, thus accommodating for planned or unplanned events prohibiting participation in a weekly session.

## Abbreviations

BMI: Body mass index; DASS-21: 21-item Depression, Anxiety and Stress Scale; DIP: Diabetes in pregnancy; FPG: Fasting plasma glucose; GDM: Gestational diabetes mellitus; GP: General practitioner; HDL: High-density lipoprotein; HOMA: Homeostasis Model Assessment; HRB: Health Research Board; HSE: Health Service Executive; IADPSG: International Association of Diabetes and Pregnancy Study Groups; IFG: Impaired fasting glucose; IGT: Impaired glucose tolerance; IR: Insulin resistance; LDL: Low-density lipoprotein; MetS: Metabolic syndrome; METS: Metabolic equivalents; MHI-5: Mental Health Index 5; NGT: Normal glucose tolerance; OGTT: Oral glucose tolerance test; PET: Pre-eclampsia toxaemia; PIH: Pregnancy induced hypertension; PP: Postprandial; QALY: Quality-adjusted life year; RCT: Randomised controlled trial.

## Competing interests

The authors declare that they have no competing interests.

## Authors’ contributions

JJI and AOD are involved in trial management, recruitment and acquisition of baseline data. JJI drafted the manuscript. PG, IG, BMcG, LGG and FPD conceived and designed the study. PG will also carry out the economic analyses and IG is involved in trial coordination. JN participated in the design of the study, performed the sample size and power calculations, and developed the plan for statistical analysis of trial outcomes. EN conducted preliminary prevalence screening for pre-diabetes and type 2 diabetes in the western regions of Ireland. BMcG and FPD revised the manuscript, and gave final approval of the version to be published. All authors read and approved the final manuscript.

## Authors’ information

JJI is a social anthropologist currently working as a Post-Doctoral Researcher in Medicine. AOD is also a Post-Doctoral Researcher in Medicine, with a background in psychology. PG is a health economist and Lecturer in the J.E. Cairnes School of Business & Economics at the National University of Ireland Galway (NUI Galway). IG is Lead Prevention Nurse and Programme Manager for Croí MyAction in Galway. BMcG is a Senior Lecturer in Clinical Psychology, Director of the Doctor of Psychological Science programme in Clinical Psychology at NUI Galway, and Joint Director of the Centre for Pain Research. LGG is a GP in practice in County Clare, Ireland, and is also founding Clinical Director of the Western General Practice Research and Education Network in Ireland and Senior Lecturer in General Practice at NUI Galway. EN is a Clinical Research Fellow at NUI Galway. JN is a Senior Lecturer in Biostatistics in the HRB Clinical Research Facility at NUI Galway. FPD is a Consultant Endocrinologist at the University Hospital Galway; Head of the School of Medicine at NUI Galway; and lead Principal Investigator responsible for the scientific and technical direction of the Atlantic DIP research programme.
